# Chronic kidney disease prevention campaign: relationship between proteinuria and elderly people

**DOI:** 10.1590/2175-8239-JBN-2022-0028en

**Published:** 2022-11-07

**Authors:** Júlio César Chaves Nunes, David Silva Camurça, Gabriel Alves Rocha, Ana Beatriz Timbó de Oliveira, Gabriela Correia Pequeno Marinho, Sérgio Gabriel Monteiro Santos, Dyego Castelo Branco Holanda Gadelha Pereira, Gdayllon Cavalcante Meneses, Elizabeth De Francesco Daher

**Affiliations:** 1 Universidade Federal do Ceará, Faculdade de Medicina, Departamento de Medicina Clínica, Fortaleza, CE, Brazil.; 2 Universidade Federal do Ceará, Departamento de Medicina Clínica, Programa de Pós-Graduação em Ciências Médicas, Fortaleza, CE, Brazil.

**Keywords:** Proteinuria, Aged, Hypertension, Renal Insufficiency, Chronic, Proteinúria, Idoso, Hipertensão, Insuficiência Renal Crônica

## Abstract

**Objective:**

To verify the relationship between the presence of proteinuria as a renal injury marker in elderly without history of systemic arterial hypertension and cardiovascular diseases. A cross-sectional study was developed from January 2014 to December 2019, through kidney disease prevention campaigns promoted by the Federal University of Ceará in the city of Fortaleza.

**Methods:**

The sample consisted of 417 elderlies. A questionnaire was used to characterize individuals and assess previous diseases, and urinalysis reagent strips were used to assess proteinuria.

**Results:**

Statistically significant differences (p < 0.05) and moderate effect sizes were found for blood pressure levels (CI 0.53-0.93), systolic blood pressure, and diastolic blood pressure (CI 0.21-0.61). Significant differences in capillary glycemia were also found between groups (p = 0.033), but with a low effect size (0.02–0.42). The group with comorbidities was 2.94 times more likely to have proteinuria than those without comorbidities (OR 2.94, CI 1.55-4.01; p < 0.05). In the group without cardiovascular disease/high blood pressure, a statistically significant association was found for previous diabetes and proteinuria (p = 0.037), presenting 2.68 times higher risk of proteinuria in those with diabetes mellitus (OR 2.68, CI 1.05-6.85). Significant association was also found between age groups, with the older group having 2.69 times higher risk of developing proteinuria (75 to 90 compared to 60 to 74 years) (CI 1.01-7.16; p = 0.045).

**Conclusion:**

Even without systemic arterial hypertension or cardiovascular disease, diabetes and older age can be considered high risk factors for proteinuria.

## Resumo

**Objetivo:**

Verificar a relação entre a presença de proteinúria como marcador de lesão renal em idosos sem histórico de hipertensão arterial sistêmica e doenças cardiovasculares. Um estudo transversal foi desenvolvido de Janeiro de 2014 a Dezembro de 2019, por meio de campanhas de prevenção a doenças renais promovidas pela Universidade Federal do Ceará, na cidade de Fortaleza.

**Métodos:**

A amostra foi composta por 417 idosos. Um questionário foi usado para caracterizar indivíduos e avaliar doenças prévias, e foram utilizadas tiras reagentes de urinálise para avaliar proteinúria.

**Resultados:**

Diferenças estatisticamente significativas (p < 0,05) e tamanhos de efeito moderados foram encontrados para níveis de pressão arterial (IC 0,53-0,93), pressão arterial sistólica e pressão arterial diastólica (IC 0,21-0,61). Também foram encontradas diferenças significativas na glicemia capilar entre grupos (p = 0,033), mas com um tamanho de efeito baixo (0,02–0,42). O grupo com comorbidades apresentou 2,94 vezes mais probabilidade de ter proteinúria do que aqueles sem comorbidades (OR 2,94; IC 1,55-4,01; p < 0,05). No grupo sem doença cardiovascular/hipertensão, foi encontrada uma associação estatisticamente significativa para diabetes anterior e proteinúria (p = 0,037), apresentando risco 2,68 vezes maior de proteinúria naqueles com diabetes mellitus (OR 2,68; IC 1,05-6,85). Também foi encontrada uma associação significativa entre faixas etárias, com o grupo mais velho apresentando risco 2,69 vezes maior de desenvolver proteinúria (75 a 90 em comparação com 60 a 74 anos) (IC 1,01-7,16; p = 0,045).

**Conclusão:**

Mesmo sem hipertensão arterial sistêmica ou doença cardiovascular, o diabetes e a idade avançada podem ser considerados fatores de alto risco para proteinúria.

## Introduction

Aging is a natural process of progressive loss of functional reserve in humans. However, excesses throughout life can lead to pathological processes^1^. Among the common conditions of aging are kidney alterations, including loss of function, cortical glomerular fibrosis, interstitial fibrosis with decrease in renal tubules, and intra-renal vascular alterations. In addition, the elderly are generally more affected by systemic diseases such as diabetes mellitus and hypertension, which are known risk factors for kidney damage and increase the prevalence of chronic kidney disease (CKD) in this population^2^.

Among the main risk factors for CKD development is high blood pressure (HBP), which is also directly associated with other risk factors, such as smoking, diabetes mellitus (DM), cardiovascular diseases, obesity, and high cholesterol^3^. The elderly are considerably affected by systemic arterial hypertension, in part due to physiological and anatomical changes that occur with age, such as reduced distensibility and increased stiffness of the great arteries^4^.

Currently, CKD is defined as abnormalities in the kidney structure or function that have been present for 3 months and have health implications; CKD is classified based on cause, glomerular filtration rate (GFR) category, and albuminuria category (CGA)^5^. Among the main markers of kidney damage are proteinuria and albuminuria. However, factors such as dehydration, urinary tract infection, alkaline urine, intense exercise, and use of phenazopyridine may interfere with the measurement of proteinuria^6,7^. CKD can cause other health problems, like mineral and bone disorder, anemia, and an increased risk of cardiovascular diseases, which increase with CKD progression^8^.

Although the relationship between cardiovascular disease (CVD) and hypertension in old age is widely known, much less is known about the extent to which proteinuria occurs in the elderly without a history of these diseases. Thus, the present study aims to verify the occurrence of proteinuria in elderly people without a history of CVD and HBP.

## Material and Methods

### Study Type

This was a cross-sectional observational study with a descriptive quantitative nature.

### Location and Time

Data collection occurred during chronic kidney disease prevention campaigns conducted from 2017 to 2019 in public spaces such as centers and parks in the twelve regions of Fortaleza, Ceará, Brazil.

### Participants

The participants were elderly of both genders, who were 60 years old or older and voluntarily visited the campaigns, totalizing 417 participants. They were consecutively attended.

### Inclusion and Exclusion Criteria

The inclusion criteria were being 60 years old or older. The exclusion criteria were not signing the informed consent form, not filling the questionnaire correctly, or not accepting the urine test.

### Ethics Criteria

The study followed the definitions of the 466/2012 resolution of the National Health Council. The research was submitted to and approved by the Research Ethics Committee of Federal University of Ceará under the consubstantiated opinion nº CAEE 78688117.0.0000.5054

### Data Collection

The data collection occurred during chronic kidney disease prevention campaigns conducted by the study’s researchers. The events took place in specific stations. The participants answered a standard questionnaire, had their blood pressure and glycemia measured, and had their urine tested by the dipstick method. The researchers organized the stations with tables and chairs and equipped each table with the necessary instruments so that the participants could follow a specific sequence. Also, the organizers provided a toilet nearby so that the participants could collect their urine.

First, the participants answered a standard questionnaire formulated by the researchers and based on the unified attendance form used by the Brazilian Society of Nephrology in its prevention campaigns^9^. The self-reported questionnaire asked about individual and family health history of arterial hypertension, diabetes mellitus, cardiovascular and renal diseases and habits such as alcohol consumption, smoking, and physical exercise. Next, the researchers measured the height, using a Sanny^®^ portable stadiometer, the abdominal circumference, using a Cescorf^®^ inelastic measuring tape, and the body weight, using a bc601^®^ scale. Then, they calculated body mass index (BMI) using BMI calculator apps.

The second and third steps consisted of verifying the participant’s blood pressure with Premium^®^ manual sphygmomanometers and Lane^®^ and Littman® stethoscopes and the capillary blood glucose with G-tech^®^ glucometers.

Finally, the participants collected their urine using plastic urine test containers, advised to despise the first stream of urine. The researchers examined the urine content using Dus10 Labor Import^®^ urinalysis test strips, which have little squares that may change color when in contact with urine, depending on the urine content. After one minute of immersion, the colors of the reagent areas are compared with reference colors and the results for protein, leukocytes, hemoglobin, glucose, and nitrite are recorded.

After data collection, and according to questionnaire responses, the participants were divided into two groups: without CVD/HBP and with CVD/HBP.

### Statistical Analysis

After data collection, the IBM SPSS 22.0 program was used to analyze the data. Kolmogorov Smirnorv and Levene tests were used to verify data normality and homogeneity, respectively. The T-test was used for comparison of independent samples. The effect size test was used to measure the power of the independent result. The Chi-Square test was used to verify the association between qualitative variables. The odds ratio (OR) was used to check the risk or protection of a group for specific exposures. A 95% confidence interval was adopted, reflecting the p < 0.05 value.

## Results

**Figure 1.**
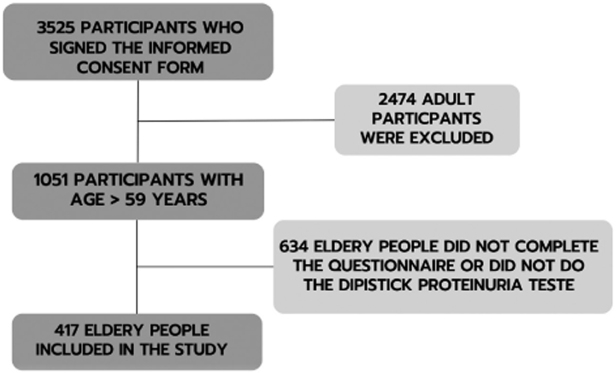
Flowchart of study participant’s inclusion procedures.

The research started with 3525 participants who signed the informed consent form. After those under 60 years old were excluded, the number decreased to 1051 participants. Nevertheless, those who didn’t complete the questionnaire or perform the dipstick proteinuria test were also excluded, leaving the final number of 417 study participants, as shown in Figure 1.

Table 1 shows the descriptive analysis of the groups divided by absence (n = 151) or presence (n = 266) of CVD/HBP, with characteristics such as age, body measurements, blood glucose, and blood pressure. Statistically significant differences (p < 0.05) and moderate effect sizes were found for systolic (CI 0.53-0.93) and diastolic (CI 0.21-0.61) blood pressure levels. Significant differences in capillary glycemia were also found (p = 0.033), but with a small effect size (0.02–0.42).

**Table 1.** Description and comparison of the groups with and without CVD/HBP

	No CVD/HBP (151)	With CVD/HBP (266)	Change (95% CI)	P value (ES)
	m ± sd	m ± sd		
Age (years)	67.75 ± 6.73	69.18 ± 7.14	−1.42 (−2.82; −0.24)	0.043^*^ (0.20)
SBP (mmHg)	129.07 ± 18.80	144.26 ± 21.67	−15.18 (−19.33; −11.03)	0.001^*^ (0.73)
DBP (mmHg)	80.27 ± 11.06	85.21 ± 12.59	−4.94 (−7.27; −2.60)	0.001^*^ (0.41)
BMI (kg/m^2^)	27.47 ± 4.27	27.61 ± 4.69	−0.137 (0.48; −1.08)	0.775 (0.03)
Abd Circumference (cm)	97.46 ± 12.22	98.74 ± 12.79	−1.28 (−3.84; 1.27)	0.330 (0.10)
CB Glucose (mg/dl)	125.95 ± 53.50	139.24 ± 64.74	−13.29 (−25.75; 0.82)	0.037^*^ (0.22)

SBP: systolic blood pressure; DBP: diastolic blood pressure; BMI: body mass index; CB: capillary blood glucose; m ± SD: mean and standard deviation; ES: effect size. Student-t test was used.

* *P*-value lower than 0.05 with a 95% confidence interval.

Table 2 shows the odds ratio for developing proteinuria in the elderly with CVD/HBP in relation to those without these diseases. The results showed that the group with comorbidities was 2.94 more likely to have proteinuria than those without it (OR 2.94, 95% CI 1.55-4.01; p < 0.05).

**Table 2:** Association and risk of proteinuria between groups

Group	Without proteinuria	With proteinuria	Adjusted OR (95% CI)	P-value
No CVD/HBP	122 (80.79%)	29 (19.2%)	2.94 (1.55-4.01)	0.001^*^
With CVD/HBP	167 (62.78%)	99 (37.21%)		

Values are reported as odds ratio and 95% confidence interval.

* P < 0.05 chi-square test.

**Figure 2.**
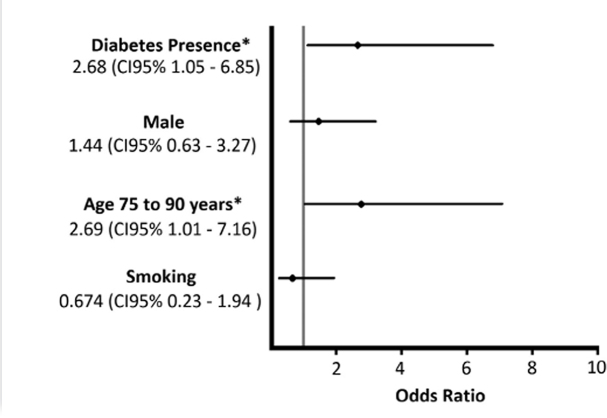
Odds ratio for the group without CVD. OR: odds ratio, CI: confidence interval, *statistically significant association obtained by the chi square test.

Within the group “No CVD/HBP”, proteinuria was associated with age, smoking, diabetes, and sex (Figure 2 and Table 3). A statistically significant association was found between previous diabetes and presence of proteinuria, with proteinuria being present in 33.33% of diabetics and in 15.70% of non-diabetics (p = 0.037), a 2.68 times higher risk of proteinuria in diabetics (OR 2.68; CI 95% 1.05-6.85). Concerning age, proteinuria was present in 16.53% of the age group 60 to 74 years and in 34.78% of the group 75 to 90 years (p = 0.045), a 2.69 times higher chances of developing proteinuria in older age group (CI 95% 1.01-7.16). The variables smoking and sex did not show a statistically significant association with proteinuria (p < 0.05).

**Table 3:** Risk of proteinuria in the group without CVD/HBP comorbidity according to the variables

*No CVD/HBP group*	Without proteinuria	With proteinuria	Adjusted OR (95% CI)	*P-value*
***Previous diabetes***				
***No***	102 (84.30%)	19 (15.70%)	2.68 (1.05-6.85)	0.037^*^
***Yes***	18 (66.67%)	9 (33.33%)		
Age				
***60 to 74 years***	106 (83.47%)	21 (16.53%)	2.69 (1.01-7.16)	0.045^*^
***75 to 90 years***	15 (65.22%)	8 (34.78%)		
Sex				
***male***	59 (76.64%)	17 (22.36%)	1.44 (0.63-3.27)	0.253
***female***	60 (83.33%)	12 (16.66%)		
Smoking				
***yes***	86 (79.63%)	22 (20.37%)	0.674 (0.23-1.94)	0.323
***no***	29 (85.3%)	5 (14.7%)		

Values reported as odds ratio and 95% confidence interval.

* P < 0.05 chi-square test.

## Discussion

A significant association was found between presence of protein in urine and age of the study subjects. In our study, elderly people aged between 75 and 90 years were more likely to have proteinuria than those aged between 60 and 74 years, even in the absence of CVD and HBP. It is important to mention that this age-adjusted model was not controlled for DM. When DM was included in the model, age was not significant. GFR decrease is part of the kidney aging process, which causes a progressive loss of nephrons as age advances, in addition to alterations on the glomerular basement membrane permeability and a modest increase on the albuminuria excretion rate. However, these processes do not seem to significantly increase mortality if proteinuria is not present^2^.

Therefore, proteinuria must be analyzed in patients with high risk of kidney disease since it is an independent indicator of CKD stage^10^. Moreover, proteinuria is a sensitive biomarker not only for kidney damage but also for cardiovascular diseases^11^. In our study, the rate of proteinuria in subjects who had previous hypertension and cardiovascular disease was significantly higher than their counterparts (p < 0.05).

For this reason, the presence of proteinuria, even if at low levels, can lead to an unfavorable prognosis. Proteinuria ≥1+ on the dipstick test was associated with a higher risk of Parkinson’s disease^12^ and higher sarcopenia^13^ incidence in old-age individuals. Similarly, in a Japanese populational study, proteinuria detected with the dipstick test was considered an independent death predictor^14^. Furthermore, patients with positive proteinuria in the dipstick test had an intermediate risk of having metabolic syndrome, hypertension, and diabetes^15^. In our study the proteinuria values were 1.55 to 4.01 times higher in the groups with CVD and HBP. Proteinuria is also pointed out as a CVD marker, which justifies the positive correlation in this study. Proteinuria was associated with myocardial ischemia, electrocardiographic indicators, higher thickness of the carotid arteries’ intima layer, left ventricular hypertrophy, and coronary artery calcification^16^.

The association between hypertension and age can be due to spontaneous vascular alterations. Natural aging processes, such as increased free radical formation, related to inflammation and oxidative stress, can cause endothelial dysfunction and higher systemic vascular resistance, leading to hypertension^17^. Additionally, unhealthy lifestyle habits, such as inadequate diet, sedentarism, smoking, excessive stress, among other factors ^17–19^ can aggravate the risk for developing high blood pressure in older people.

Hypertension is both a cause and an effect of CKD, contributing to renal function decrease. As the glomerular filtration rate reduces, the incidence and gravity of hypertension grow. Also, hypertension and CKD are independent risk factors for cardiovascular disease^20^. HBP treatment is considered fundamental for delaying CKD progression and reducing cardiovascular risk^21^. In a study of elderly people from the same location as the present study, a higher CKD incidence was observed in men, and it was related to incorrect use of antihypertensive drugs, leading to DBP increase^22^. However, in this study, no significant difference was found between male and female groups regarding the presence or absence of proteinuria (p = 0.253).

Recent studies^17,18^ also associate older age and BMI with higher chances of developing high blood pressure. A long term prospective study^18^ with 1132 participants showed that men with normal BMI in early adulthood who became overweight/obese in midlife had twice the risk of developing hypertension than those that maintained the expected BMI. The study also states that even a moderate weight gain can be associated with a notorious risk of having HBP.

The direct relationship between capillary blood glucose and high blood pressure is not well explored in the literature. This method of blood glucose measurement is not capable to test whether hyperglycemia is a constant and probably pathological condition if only an isolated measure is analyzed, since it indicates the blood sugar level at the time of the exam. Nevertheless, considering that the test is for a chronic condition, a potential association with hypertension can be mediated by the endothelial dysfunction in both pathologies. The vascular endothelia has several functions, including maintenance of vascular tone and consequently blood pressure through synthesis of vasodilator and vasoconstrictor substances. Chronic hyperglycemia can induce an increase in oxidative stress, which is associated with endothelial dysfunction^23,24^. Moreover, hyperglycemia is related to insulin resistance and hyperinsulinemia, which also has a role in endothelial dysfunction development. Considering that dysfunction is an altered execution of functions, hyperglycemia can be a cause or a consequence of several diseases, such as insulin resistance itself and hypertension^23,25^.

Hyperglycemia has been associated with higher risk of diabetic nephropathy and CKD development and progression. DM and CKD are cardiovascular risk factors and have a synergistic effect in raising cardiovascular mortality. The contribution of diabetes as a cause of advanced CKD has increased, with 40% of new dialysis patients have kidney disease attributable to diabetes^26^. In this study, significant values of proteinuria were found in diabetics even without other comorbidities (p = 0.037). However, the groups with HBP and CVD also had higher capillary blood glucose levels (p = 0.037). Thus, this study showed a significant relationship between blood glucose levels and proteinuria, either alone or associated with HBP and CVD.

Smoking is related to higher CKD risk, independent of other well-established risk factors, such as age, HBP, and DM. Nevertheless, our analysis did not show statistical significance in the association between smoking habit and proteinuria, similar to another study that also did not observe an association between smoking and albuminuria/proteinuria incidence in the general adult population. The pathology of renal damage caused by smoking is not well understood. However, smoking has some chronic effects like endothelial dysfunction, inflammation, oxidative stress, glomerulosclerosis, and tubular atrophy^27^. Moreover, smoking is related to insulin resistance increase^28^, which is associated with a decrease in glomerular filtration rate^29^.

One of the study limitations was the use of the dipstick test as a early detection method of urine protein since the gold standard is the estimated glomerular filtration rate, obtained by serum creatinine results and using the Chronic Kidney Disease Epidemiology Collaboration (CKD-EPI) formula. Also, other kidney damage markers can be used, such as plasma creatinine, which is the endogenous marker that better resembles the profile of an endogenous substance for measuring the glomerular filtration rate. Another limitation was the study format that used self-reported comorbidity, which may limit the interpretation and response power of the results.

## Conclusion

We concluded that diabetes can independently and dramatically increase the risk of urinary protein, an important CKD biomarker, in elderly without systemic arterial hypertension or cardiovascular disease. Another important finding was that despite not adjusting for DM, the risk for proteinuria increases from the age of 75, even without CVD or HBP, which requires greater attention in this age group. Increase prevention of DM, CVD, and HBP can result in significant protection against proteinuria, thereby reducing the risk for CKD and increasing quality of life in this population.
